# Botulinum Toxin A for Controlling Obesity

**DOI:** 10.3390/toxins8100281

**Published:** 2016-09-26

**Authors:** Raffaela Pero, Lorena Coretti, Francesca Lembo

**Affiliations:** 1 Department of Molecular Medicine and Medical Biotechnology, University of Naples “Federico II”, Naples 80131, Italy; lorena.coretti@tiscali.it; 2 Dipartimento di Farmacia, Università degli Studi di Napoli “Federico II”, via D. Montesano 47, Naples 80131, Italy; frlembo@unina.it

**Keywords:** botulinum toxin A, obesity, intragastric injection

## Abstract

Rapid growth of the overweight population and the number of obese individuals in recent decades suggests that current strategies based on diet, exercise, and pharmacological knowledge are not sufficient to address this epidemic. Obesity is the result of a high caloric intake and energy storage, not counterbalanced by an equally important energy expense. Botulinum toxin type A (BoNT-A) use is rapidly expanding to include treatment of a variety of ophthalmological, gastrointestinal, urological, orthopedic, dermatological, secretory, painful, and cosmetic disorders. Many studies evaluating the effect of BoNT-A in gastric antrum e/o fundus for the treatment of obesity have been published. This treatment modality was based on the observation that gastric injection of BoNT-A in laparatomized rats induced a significant reduction of food intake and body weight. These studies have been published yielding debated results. Differences in the selection of patients, the doses of BoNT-A, the method of administration of the toxin, and the instruments of evaluation of some parameters among these studies may be the cause. In this review, it will study the state-of-the-art use of BoNT-A in obesity basic science models and review the clinical evidence on the therapeutic applications of BoNT-A for obesity.

## 1. Introduction

Obesity is fast emerging as one of the greatest challenges of human health. The number of obese people has nearly doubled since 1980. According to the World Health Organization (WHO) there are currently about 1.5 billion overweight adults in the world and, of these, 500 million are obese. In addition, about 42 million children under five years old are overweight or obese.

The situation in the US is quite alarming, with 65% of US adults overweight or obese. Obesity is a risk factor for a large number of diseases: cardiovascular, metabolic such as type 2 diabetes, and many forms of cancer [1].

At the biological level, obesity results from an imbalance of energy balance in the body. If the input of energy (introduced with food) consistently exceeds energy expense, the extra energy is transported and stored in the form of triglycerides [2]. A large number of factors, namely genetic, environmental, behavioral, biological, and lifestyle factors, play an important role in increasing food consumption and developing overeating disorders [3].

Obesity is defined by body mass index (BMI), which is obtained by dividing a person’s weight by the square of the person’s height. The BMI does not consider the distribution of body fat, and, because of the relationship between abdominal obesity with cardiovascular disease (CVD) and other metabolic risk factors in clinical practice, it is useful to measure, in addition to BMI, the waist-hip ratio (WHR). The WHR is the ratio of the circumference of the waist to that of the hips and is calculated as the waist measurement divided by the hip measurement (*W*/*H*) [4,5].

Unfortunately, the traditional therapies based on diet, exercise, behavior modification, and medication have had little effect, especially in severely obese people. Surgical approaches provide nowadays the most effective treatment of obesity but present possible risks. Only 1% of patients are estimated to benefit from bariatrics [6].

The burgeoning field of endoluminal therapy allows us today to consider even less invasive 0procedures to reach a broader population of obese patients. 

Botulinum neurotoxins, produced by the bacterium *Clostridium botulinum*, have been widely applied medically since the first use of BoNT-As for ocular strabismus [7]. In 1993, Pasricha et al. reported on the use of BoNT-A in the gastrointestinal (GI) tract [8], demonstrating that BoNT-A could be safely injected into the smooth muscle of the GI tract and that it decreased resting pressure of the lower esophageal sphincter (LES) in piglets. This novel study opened up an entirely new field of research and therapy for many GI motility disorders.

Nowadays, BoNT serotype A is the most widely used clinically, as it can inhibit the muscular contractions of smooth and striated muscles [9], and this property has been used in the treatment of some digestive illnesses [10,11]. Gui et al. was the first to show that intramuscular injections of BoNT-A in the gastric wall of laparatomized normal-weight rats significantly reduced their food intake and body weight [12]. In this prospective trial, they measured daily food intake over 7 weeks and body weight over 10 weeks in three groups of rats: a control group (no treatment; *n* = 5), a laparotomy group with antral BoNT-A injection (20 IU; *n* = 14), and a laparotomy group with saline antral injection (*n* = 14). They found that BoNT-A injection led to a significant reduction in both food intake (*p* < 0.05) and weight (*p* < 0.001) compared with the sham injection group. Subsequently, similar findings were confirmed by Coskun et al. in obese rats [13]. In humans, injections with BoNT-A has led to conflicting results, probably coming from differences in the sites of administration (antrum and/or fundus region), the doses of the toxin, and patient selection [14]. Very recently, in a meta-analysis and meta-regression of eight studies, Bang et al. analyzed a total of 115 patients (79 treated vs. 36 placebo). Wide area injection including the fundus or body rather than the antrum only and multiple injections (>10) were associated with weight loss [15].

De Moura et al. [16] assessed a systematic review to evaluate the meta-analysis performed by Bang et al. [15] using a methodology based on the guidelines from the Preferred Reporting Items for Systematic Reviews and Meta-Analyses (PRISMA) statement and the Assessment of Multiple Systematic Reviews (AMSTAR) [17,18]. They found that serious aspects compromise the reliability, consistency, and applicability of the recommendations about the use of botulinum toxin A in the treatment of obesity, concluding that the meta-analysis of Bang et al. [15] is the weakest form of evidence and has no power to support any recommendation [19,20].

This review discusses the available mechanism and current medical evidence of intragastric BoNT-A injection for treating obesity.

## 2. Botulinum Toxin

BoNT-A is a neurotoxic protein produced by Gram-positive, anaerobic bacterium *Clostridium botulinum* [21]. There are seven different serotypes of botulinum toxins designated by the letters A, B, C1, D, E, F, and G. Each serotype has more subtypes (e.g., the subtype A contains four distinct subtypes). All serotypes have a similar chemical structure and are neurotoxins, with the exception of the C2 subtype [22,23]. Recently, a new serotype (BoNT/H) has been proposed but has yet to be validated [24].

Botulinum toxin acts in the neuromuscular junction (endplate) blocking the release and the effects of acetylcholine, an acetic acid ester and choline, responsible for neurotransmission in the central nervous system (CNS) and peripheral nervous system (PNS) [25]. 

Botulinum toxin is a metal-protein with endopeptidase activity. The metal in question is zinc. The general structure presents two chains of a total weight of approximately 150 kDa. They are a heavy chain (H, 100 kDa) and a light chain (L, 50 kDa) joined by a disulfide bridge. The heavy chain is in turn constituted from the *N*-terminal domain (HN) and the C-terminal domain (HC). Each botulinum toxin is initially synthesized as a double polypeptide, but the biological activity requires a post-translational proteolysis that cleaves the polypeptide in two separate portions [26]. In nature, the botulinum toxin exists as complex consisting of a protective coating of catalytically inactive proteins, called “proteins associated with neurotoxins” (NAPs, neurotoxin-associated proteins). The NAPs are a set of five proteins: (i) four different hemagglutinins (HP, hemagglutinizing proteins) and (ii) one non-hemagglutinin (NHP, Non-hemagglutinizing protein). The NAPs are synthesized by *Clostridium* and defend the neurotoxin from possible destruction by the gastric activity of the stomach. However, the presence of the NAPs involves the occurrence of severe and frequent immune responses and a greater difficulty in obtaining a precise dosage of the toxin [27]. 

The two chains that constitute the molecular organization of BoNT-A have different functions. The heavy chain binds to a specific receptor present on the cell membrane of the synaptic button, the synaptic vesicle glycoprotein 2 (SV2 receptor). This binding starts an invagination of the membrane, and a consequent endocytosis that makes able the botulinum toxin to penetrate inside the synaptic button. The light chain is a protease and performs the catalytic function of the toxin. The mechanism of action of BoNT-A is based on three key events: (i) binding to receptors; (ii) internalization and translocation into the cytoplasm; and (iii) enzymatic modification of the target [28].

### 2.1. Binding to Receptors

Neuromuscular cholinergic synapse is a structure used for the transduction of motor stimulation by the nervous system and muscle apparatus of an organism. Its main function is to translate an electrical pulse, coming from the neuronal axon, in the chemical stimulus by means of the release of the neurotransmitter acetylcholine.

When the action potential reaches the synaptic terminal, there is an influx of calcium (Ca^++^), due to the opening of voltage-gated ion channels, from the extracellular environment. This ion, interacting with various synaptic proteins, determines the fusion of vesicles containing the neurotransmitter, causing the release of the latter (Ach) [29]. 

In the synaptic cleft, acetylcholine will bind to nicotinic cholinergic receptors of the postsynaptic region. These receptors are ion channels that bind two molecules of Ach and undergo a conformational change determining opening channels. At this point, there is an ionic influence, at the muscular level, which leads to the mobilization of Ca^++^ from intracellular stores with a consequent contraction of muscle fibers. In the body fluids, the botulinum toxin is able to reach the muscle junctions that, following its internalization, will carry out the toxic activity adapted to block the transmission of nerve impulses [30]. 

### 2.2. Internalization and Translocation into the Cytoplasm

Neurotransmitters are small molecules of a different nature that are released from neurons and are able to evoke a response in a postsynaptic element. They are not dissolved in the cytoplasm but are accumulated in special vesicles of synapses. After the action of the potential axon, the fusion of the vesicles occurs with a consequent release of the molecules contained therein. This process is not spontaneous, nor left to chance. There is, in fact, a fine tuning of all the processes that lead to neuroexocytosis: the mobilization of vesicles, fusion with the cell membrane, and subsequent endocytosis for the recovery of the functionality [31].

The neurotransmitter is accumulated in the vesicles lumen through a specific carrier that uses the H^+^ gradient created by ATPase activity of a proton pump. Many of the vesicles at synaptic terminals are anchored to actin cytoskeleton through interactions regulated by proteins such as synapsin. They should therefore be freed from this constraint in order to reach the region that is “active” of synapses. After mobilization, the vesicles fuse with the membrane through various processes. In the tethering process, the vesicles are able to approach and interact with the presynaptic membrane in special zones called “active zones” [32]. The docking process allows the vesicles to binds to the plasma membrane forming the SNARE complex (SNAPs-receptor). This complex consists of three proteins: syntaxin (cell membrane), SNAP-25 (cell membrane), and VAMP/synaptobrevin (vesicle membrane) [33].

During the priming process, the vesicles mature and are able to fuse with the cell membrane perceiving only one activating stimulus. At this point, the release of calcium induced by an action potential is able to melt the vesicle membrane with the cell, thus permitting the outflow of neurotransmitters. After this step, the vesicles must be endocitate to regain functionality. The SNARE complex serves as a receptor for the cytosolic factors a, b, and g SNAPs (synaptosomal associated proteins), which, in turn, mediate the interaction with the NSF (*N*-ethylmaleimide-sensitive fusion protein). At this point, the ATPase is able to dissociate the complex, and the vesicles can be retrieved via endocytosis. The endocytosis is characterized by the formation of a coating of clathrin and by the action of a dynamin, GTP-dependent protein, closing the fusion of the neck vesicles [34].

### 2.3. Enzymatic Modification of the Target

SNARE is a trimeric complex composed of the proteins: VAMP/synaptobrevin, SNAP-25, and syntaxin. Depending on their location, these proteins are classified as v-SNARE (vescicle) if associated with the vescicular membrane, or t-SNARE (target) when associated with the cytoplasmic. VAMP (vescicle associated membrane protein) is a protein of 13 or 23 kDa (VAMP-1 and VAMP-2 are neuronal isoforms). VAMP is anchored to the vescicular membrane by the carboxyl terminal, while the amine terminal is completely in the cytosol. VAMP is also involved in processes of exocytosis of non-neuronal cells [35,36].

SNAP-25 (synaptosomal associated protein of 25 kDa) contains 206 amino acids. The peculiarity of this protein resides in the absence of intramembrane segments. Its anchoring is due to the palmitoylation of four cysteines located in the center of the protein chain. There are several isoforms of SNAP-25, for example, SNAP-29 and SNAP-23; all are involved in the regulation of exocytosis phenomena [37].

Syntaxin is a plasma membrane protein of about 35 kDa that presents a transmembrane segment (projecting the synaptic space) near the carboxyl terminus. This protein, in addition to binding to VAMP and SNAP-25, also interacts with Synaptotagmin, a calcium-sensitive protein, involved in the vesicular membrane fusion and, therefore, in the release of neurotransmitter (SNAP-25 is also able to interact with Synaptotagmin) [38]. Crystallography studies have enabled an understanding of how various proteins interact with each other for the formation of the SNARE complex. It is composed of four parallel helices (two of which belong to SNAP-25) tightly wound on themselves to form a very stable complex. The proteins of the SNARE complex are targets of botulinum toxins. The cutting of these proteins may result in a failure pairing of the same or not very stable interaction that will give rise to a SNARE complex non-functional or however scarcely efficient. It was shown that the BoNT-As are not able to perform their proteolytic action on complexed protein, but only when they are in free form [39]. 

BoNT-A acts on a pre-synaptic level, blocking the release of the neurotransmitter acetylcholine at the neuromuscular junction. Acetylcholine is the neurohormone that allows the SNC to communicate with the muscle cells stimulating the striated determining muscle contraction, and allows the SNA to communicate with the sweat glands stimulating their secretion. The different serotypes of botulinum toxin have specific molecular targets: the toxin A catalyzes the cleavage of SNAP-25 molecules, the toxin B catalyzes the cleavage of VAMP, the serotypes D, F, and G also have VAMP as a substrate, and the serotypes C1 and E have SNAP-25 as a substrate [40]. 

The cleavage of these proteins destroys the formation of functional SNARE complexes, preventing the adhesion and the fusion of the vesicles with the presynaptic membrane and inhibiting the exocytosis of acetylcholine. In this way, the signal conduction of cholinergic neurons is interrupted. The various serotypes have the ability to specifically hydrolyze a single peptide bond of these proteins, and there is a kind of structural recognition of targets by the botulinum toxins. BoNT-A also acts on other nerve endings. In fact, it was observed, in preclinical studies, that BoNT-A reduces pain by inhibiting the neurotransmission of substance P, glutamate, and calcitonin gene-related peptide. The proteins of the SNARE complex play a key role in the release of acetylcholine, promoting the fusion of the synaptic vesicle membrane in which the acetylcholine is stored with the synaptic membrane button [41].

SNAP-25 is hydrolyzed, and this event makes the fusion between the membrane of synaptic vesicles and the synaptic button impossible. In this way, the acetylcholine can not be released in the synaptic cleft determining the characteristic flaccid paralysis of the treated muscles. Later, in a few days, the sprouts appear (nerve germination); at 28 days, they will have freed the acetylcholine. This production is, however, insufficient for a regular decrease. In 2–3 months, it can enjoy a muscle recovery for the release of an effective concentration of acetylcholine. Meanwhile, the production of SNAP-25 continues. In 3–6 months, the plaque resumes its normal function, while the sprouts disappear [41]. 

## 3. Mechanisms of BoNT-A in the GIT Muscle

There have been several studies showing that BoNT-A inhibits the vesicular neurotransmitter release from neurons and is widely used as a therapeutic agent for a variety of spastic muscular disorders including those involving visceral muscle [42,43]. BoNT-A acts to inhibit acetylcholine release at the neuromuscular junction, hypothetically delaying gastric emptying and inhibiting ghrelin secretion, a potent hormone released from the gastric fundus that stimulates hunger [44]. Although BoNT-A can clearly inhibit the release of acetylcholine, little else is known about its effects in the GIT (Gastrointestinal) muscle. In gastrointestinal smooth muscle, botulinum toxin appears to also reduce cholinergic transmission by inhibiting Ach release, as shown in in vitro [8] and in vivo studies [45,46]. SNAP-25, the substrate for botulinum toxin, is also present in gastrointestinal smooth muscle, suggesting an additional site for botulinum toxin [47]. 

James et al. [48] demontrated that BoNT-A has a dual effect on gut smooth muscle tone, with low concentrations (2 IU/mL) inhibiting neurally mediated contractile responses and higher concentrations (10 IU/mL), directly inhibiting smooth muscle contractility in response to exogenous Ach. Enteric motor neurons can be considered either excitatory or inhibitory on smooth muscle tone; thus, excitatory motor neurons contain both Ach and the tachykinin substance P (SP) [49,50]. Blocking both Ach and SP release from enteric neurons, BoNT-A induces paralysis of gastrointestinal smooth muscle not only because of a lack of Ach but also from the lack of endogenous SP that is required to maintain sensitivity to exogenous contractile agonists. This study demonstrated a novel role for coneurotransmission in the enteric nervous system with important physiological and clinical implications [51]. SP depolarizes the membrane potential inducing contraction in gastrointestinal smooth muscle. It is an undecapeptide belonging to the tachykinin family and can induce strong contractions in pylorus via neurokinin 1 receptor (NK1R), the preferred receptor for SP [52,53]. Experimental evidence suggests that BoNT-A directly inhibits SP-induced pyloric smooth muscle contractility in a concentration- and time-dependent manner [54]. 

The effects of BoNT-A are time and concentration dependent as axonal sprouting and accumulation of extrajunctional Ach lead to slow reversal of denervation [55]. BoNT-A has been found to be effective in the treatment of spastic disorders of smooth muscle in the upper and lower gastrointestinal tract. Case reports and prospective trials have shown positive results with BoNT-A administration in the treatment of diffuse esophageal spasm, achalasia, oropharyngeal dysphagia, anismus, anal fissures, and anterior rectocele [56,57,58,59,60,61]. 

## 4. Clinical Studies of Intragatsric BoNT-A for Obesity

Several studies have supported the clinical use of the BoNT-A injection into the gastric antrum in obese patients. 

Rollnik et al. demonstrated the results of BoNT-A gastric antrum injection in treating obesity, and they showed a reduction in body weight of 9 kg and 32.5% of the caloric daily intake four months after treatment [62].

García-Compean et al. performed, by endoscopy, prepyloric antral gastric wall injection with 100 UI of BoNT-A in 12 obese patients, and they evaluated body weight and solid gastric emptying before, and 4 and 12 weeks after, treatment. The results demonstrated that body weight and gastric emptying did not show significant changes compared with baseline values. Abnormal gastric emptying (solid gastric retention at 90 min > 50%) was observed in 22% of patients after 4 weeks and in 25% after 12 weeks [63]. In another pilot study, Albani et al. [64] analyzed the efficacy of 500 UI endoscopic BoNT-A injections to the gastric antral region in eight patients with severe obesity and multiple dietary treatment failures. They observed no clinically significant side effects and a reduction in body weight at one month, independently of a specific diet. At four months, three of the patients demonstrated further weight loss. 

Junior et al. reported their experience of different doses (200–300 IU) of antropyloric region BoNT-A injection, at different sites, in 12 patients with class III obesity, and body weight and gastric emptying time did not reduce significantly before and after injection over a period of 12 weeks. All patients reported a feeling of early satiety [65]. In a randomized double blind controlled trial, with 14 obese patients assigned to three groups in which Btx-A at 133 IU, Btx-A at 200 IU, and saline at 8 mL was injected into the gastric angulus in eight sites around the gastric antral circumference, all male patients (8 patients), both in the BTX133 and BTX200 groups, reported weight loss, which was not statistically significant. In the saline group, only one male patient lost weight. The effects on gastric emptying were variable. Most of the BoNT-A-treated patients reported a reduced appetite [66]. A double blind controlled study evaluated the responses of 24 morbidly obese patients at 200 IU BoNT-A or a placebo into the antrum and fundus of the stomach by intraparietal endoscopic administration. All patients treated with BoNT-A had significantly greater amounts of weight loss after 8 weeks. In addition, this study found a prolonged gastric emptying time and reduced maximal gastric capacity for liquids [67]. Similar results have been found in an open label study of 10 obese adults who received 100 units (4 patients) or 300 units (6 patients) of BoNT-A and were followed for 16 weeks [68].

A randomized controlled trial by Li et al. [69] in 20 obese patients demonstrated statistically significant weight loss, ranging from 1 to 12 kg and decreased triglyceride levels in those injected with BoNT-A. Gastric emptying times were longer, and a decrease in fasting ghrelin levels was appreciated. However, a larger randomized placebo controlled trial by Topazian et al. [70], who enrolled 60 obese patients in a six-month trial, demonstrated a delay in gastric emptying without the effects of early satiety, in altered eating behaviors, or in weight loss. In this study, only antral injections were performed as opposed to both antral and fundal injections. These results may reflect the relatively short duration of action of BoNT-A and certainly indicate the need for further evaluation of this technique as a method for weight loss. In summary, differences among several open-label studies and double-blind, placebo-controlled trials have been reported (Table 1).

## 5. Conclusions

In summary, given the minimal adverse events noted in studies and the widespread availability, BoNT-A has the potential to have a large role in the treatment of obesity. However, additional investigation into the sites of BoNT-A injection and the optimum dosage are necessary. One major limitation with BoNT-A is its relatively short duration of action, which is approximately three to six months. Furthermore, data regarding long-term outcomes is lacking and additional studies may be warranted. The mechanisms of BoNT-A treatments include centrally mediated alterations of appetite or satiation, and the alteration of stomach capacity, gastric emptying, or incretin hormones. BoN-TA inhibits acetylcholine release at the neuromuscular junction, hypothetically delaying satiation, and appetite. Neurotransmission in the gastrointestinal (GI) tract relies on several mediators. Acetylcholine represents the most important stimulating mediator both of the intrinsic (myoenteric) and extrinsic (vagal) nervous systems [71]. Botulinum toxin A binds with high affinity to cholinergic nerve endings and selectively inhibits their activity [72]. Cholinergic neurons are localized in different brain regions, and activation of their receptors has important roles in the regulation of various appetitive behaviors, in part through their interactions with mesolimbic dopamine (DA) systems. The existence of a cholinergic interneurons activity in the nucleus accumbens (NAc) and cholinergic projections to the ventral tegmental area (VTA), which function to affect feeding behavior, have been demonstrated. These output pathways may have distinct roles in promoting either satiety or appetite, depending on their specific co-transmitters [73]. DA promotes the appetite or satiety through activation through activation of a select GABA output pathway and cholinergic activation of these pathways via muscarinic receptors. Behavioral studies support the theory that increasing levels of accumbens ACh can promote satiety. In a very recent study, it has been demonstrated that increased extracellular ACh in the NAc can inhibit feeding. Thus, by promoting ACh levels in the synapse, food intake is attenuated [74].

Future treatment may be individualized based on quantitative GI and behavioral traits measured in obese patients. Additional studies are needed to assess the role of botulium toxin in the treatment of obesity.

## Figures and Tables

**Table 1:** Botulinum toxin (BoNT/A) for the treatment of obesity.

Authors	Study	Patients	BoNT/A (IU)	Injections Place	Results
Rollinik et al., 2003 [62]	Open label	1	100	Antrum	Reduction of body weight and 32.5% of the caloric daily intake at 4 months
Garcia-Compean et al., 2005 [63]	Open label	12	100	Antrum	Body weight and gastric emptying did not show significant changes at 12 weeks
Albani et al., 2005 [64]	Open label	8	500	Antrum	Reduction of body weight at 1 month
Junior et al., 2006 [65]	Open label	12	200–300	Antrum	Body weight and solid and gastric emptying did not differ significantly at 12 weeks
Gui et al., 2006 [66]	Double-blind, randomized, placebo-controlled	14	133–200 vs. saline	Antrum	Weight reduction not statistically significant, variable gastric emptying and reduced appetite at 5 weeks
Foschi et al., 2007 [67]	Double-blind, randomized, placebo-controlled	24	200 vs. saline	Antrum/Fundus	Significantly weight loss, reduction in maximal gastric capacity for liquids and prolongation in gastric emptying time at 8 weeks
Topazian et al., 2008 [68]	Open label	10	100–300	Antrum	Reduction of body weight loss and prolongation in gastric emptying at 16 weeks
Li et al., 2012 [69]	Randomized controlled	20	200–300	Antrum	Significant reduction of body weight and gastric emptying levels at 12 weeks
Topazian et al., 2013 [70]	Double-blind, randomized, placebo-controlled	60	100–300–500 vs. saline	Antrum	Delay in gastric emptying without the effects of early satiety, altered eating behaviors, or weight loss at 6 months
